# Factors associated with dropout in a long term observational cohort of fishing communities around lake Victoria, Uganda

**DOI:** 10.1186/s13104-015-1804-6

**Published:** 2015-12-24

**Authors:** Andrew Abaasa, Gershim Asiki, Juliet Mpendo, Jonathan Levin, Janet Seeley, Leslie Nielsen, Ali Ssetaala, Annet Nanvubya, Jan De Bont, Pontiano Kaleebu, Anatoli Kamali

**Affiliations:** Uganda Research Unit on AIDS, Medical Research Council/Uganda Virus Research Institute (MRC/UVRI), P.O Box 49, Entebbe, Uganda; Uganda Virus Research Institute/International AIDS Vaccine Initiative (UVRI/IAVI)-HIV Vaccine Program, Entebbe, Uganda; London School of Hygiene and Tropical Medicine, London, UK; International AIDS Vaccine Initiative, New York, USA; Faculty of Health Sciences, School of Public Health, University of the Witwatersrand, Johannesburg, South Africa

**Keywords:** HIV, Long term, Dropout, Migration, Fishing communities, Lake Victoria Uganda

## Abstract

**Background:**

Fishing communities are potentially suitable for Human immunodeficiency virus (HIV) efficacy trials due to their high risk profile. However, high mobility and attrition could decrease statistical power to detect the impact of a given intervention. We report dropout and associated factors in a fisher-folk observational cohort in Uganda.

**Methods:**

Human immunodeficiency virus-uninfected high-risk volunteers aged 13–49 years living in five fishing communities around Lake Victoria were enrolled and followed every 6 months for 18 months at clinics located within each community. Volunteers from two of the five communities had their follow-up periods extended to 30 months and were invited to attend clinics 10–40 km (km) away from their communities. Human immunodeficiency virus counseling and testing was provided, and data on sexual behaviour collected at all study visits. Study completion was defined as completion of 18 or 30 months or visits up to the date of sero-conversion and dropout as missing one or more visits. Discrete time survival models were fitted to find factors independently associated with dropout.

**Results:**

A total of 1000 volunteers (55 % men) were enrolled. Of these, 91.9 % completed 6 months, 85.2 % completed 12 months and 76.0 % completed 18 months of follow-up. In the two communities with additional follow-up, 76.9 % completed 30 months. In total 299 (29.9 %) volunteers missed at least one visit (dropped out). Dropout was independently associated with age (volunteers aged 13–24 being most likely to dropout), gender [men being more likely to dropout than women [adjusted hazard ratio (aHR) 1.4; 95 % confidence interval (CI) 1.1–1.8)], time spent in the fishing community (those who stayed <1 year being most likely to dropout), History of marijuana use (users being more likely to dropout than non-users [1.7; (1.2–2.5)], ethnicity (non-Baganda being more likely to dropout than Baganda [1.5; (1.2–1.9)], dropout varied between the five fishing communities, having a new sexual partner in the previous 3 months [1.3 (1.0–1.7)] and being away from home for ≥2 nights in the month preceding the interview [1.4 (1.1–1.8)].

**Conclusion:**

Despite a substantial proportion dropping out, retention was sufficient to suggest that by incorporating retention strategies it will be possible to conduct HIV prevention efficacy trials in this community.

**Electronic supplementary material:**

The online version of this article (doi:10.1186/s13104-015-1804-6) contains supplementary material, which is available to authorized users.

## Background

In 2012, an estimated 2.3 million people worldwide became newly infected with human immunodeficiency virus (HIV) and the majority of these new infections occurred in sub-Saharan Africa [1] despite available behavioural and biomedical prevention strategies. The best long-term hope for controlling the human immunodeficiency virus/acquired immunodeficiency syndrome (HIV/AIDS) pandemic is a safe, effective and affordable preventive vaccine [2]. A number of HIV vaccine preparedness cohorts have been established in sub Saharan Africa to describe the HIV epidemiology and to assess suitability and willingness to participate in future HIV vaccine and other prevention trials [3–5]. In order to provide sufficient statistical power, these cohorts need to have a high incidence of HIV, recruit volunteers who are able to be traced and who can complete all study visits [6].

Attrition from longitudinal cohorts poses a challenge as it could bias the estimates of outcome measures and reduce the statistical power. Completion of all study visits by as many volunteers as possible is regarded as an essential component of intervention or observational cohort studies [7]. Volunteers who drop out of a study might have different characteristics from those who complete all study visits. This could result in either under- or over-estimation of the incidence of the outcome measure. Information is therefore needed on study dropout to inform future design, study inclusion/exclusion criteria and adherence counseling strategies in order to retain as many volunteers as possible. Little is known about long-term retention among fishing populations in longitudinal studies. A 1 year study in the fishing communities further North of Lake Victoria indicated a completion rate of 76.9 % [8]. In the same study, follow up rates were significantly lower among volunteers aged 18–24 years, non-Baganda ethic groups, unmarried participants, people working in a bar/lodge/restaurant and those with less than 1 year’s stay in the fishing communities. In a neighbouring non-fishing population a rural community vaccine preparatory cohort (VPS) indicated age and reported condom use as the only factors associated with volunteer cohort dropout [9]. Therefore robust data is needed on long term follow-up and retention of high risk populations to guide design of future efficacy trials in these populations.

Since 2006, the Medical Research Council/Uganda Virus Research Institute (MRC/UVRI) Uganda Research Unit on AIDS, in collaboration with the Uganda Virus Research Institute/International AIDS Vaccine Initiative (UVRI/IAVI) HIV Vaccine Program (Entebbe, Uganda) has been conducting vaccine preparatory studies (VPS) to identify potential populations for future HIV vaccine efficacy trials [5, 9, 10]. As part of this, in 2009 a European and developing countries clinical trials partnership (EDCTP) study was initiated in five fishing communities in Uganda to assess the suitability of fishing communities for future HIV prevention trials. This study found an HIV incidence of 4.9 per 100 person-years at risk [11]. Similarly, the rates of HIV in fishing communities throughout the African Great Lakes region are consistently higher than in surrounding agricultural areas [12]. Fisher-folk in this region have been described as among the most vulnerable to HIV infection [12]. The high HIV incidence in these communities makes them suitable for future prevention trials; however there is uncertainty around the feasibility of including these populations in efficacy trials because of the perception that fisher-folk are highly mobile and less likely to comply with all study procedures and visits. The objective of this analysis was to determine the dropout rate and find factors associated with dropout from a long term fisher-folk cohort in order to inform the design of future HIV prevention trials.

## Methods

### Study sites

Five fishing communities situated on the shores of Lake Victoria in Uganda were selected using pre-defined criteria, mapped and a census carried out, and identification numbers assigned to all residents and regular visitors. The selection criteria for the communities included; location within a distance of 50 km from the research centres, legal status (gazetted by the Uganda Fisheries Department), population (at least 1000 adult residents). The fishing communities selected are spread across three lakeshore districts (Masaka, Wakiso, and Mukono). Masaka and Wakiso districts had two communities each on the shores while the Mukono district community is an island about 40 km by boat from the Wakiso communities.

### Study recruitment

The five communities had a total population of 15,415 people, of whom 10,188 (66 %) were 13–49 years old. All community members aged 13–49 years were given cards bearing their identification number (assigned through the census) and invited for screening for HIV at designated study clinics established in the fishing communities. Volunteers were requested to give their consent to participate (informed written consent was obtained from adults aged ≥18 years and assent in conjunction with parent/guardian consent for minors aged <18 years) in an incidence cohort study. Volunteers were tested for HIV and those found negative were requested to enrol into the cohort. Enrolment was carried out consecutively giving more quotas to larger fishing communities basing on population size until 1000 HIV negative high risk individuals were identified and enrolled. The sample of 1000 was pre-determined and was expected to accumulate at least 1200 person years needed to estimate the HIV incidence rates of 5/100 person-years at risk (PYAR) with a precision of ± 1.2. The sample of 1000 volunteers was accrued after 2074 individuals had been screened. High risk for HIV was defined as self-report of any of the following in the previous 3 months: unprotected sex with one or more sex partners, history of sexually transmitted infections (STIs), knowledge of HIV-positive partner and being away from home for ≥2 nights per month. Volunteers who were HIV positive during screening were referred to HIV service providers. Approximately half were selected from the two Masaka District communities, and half from the other three communities.

### Cohort follow-up

Volunteers who met the criteria of high risk and were enrolled and followed-up every 6 months between 2009 and 2011 at the study clinics located in each of the fishing communities. Human immunodeficiency virus counseling and testing was provided and data on sexual behaviour collected during all visits. They were also offered treatment for other sexually transmitted infections (STIs). Volunteers who missed study visits were reminded at least twice within 2 days of the missed visit by a community mobiliser initially by phone contact, followed by a home visit. Volunteers who could not be traced were considered lost to follow up. The full cohort details for the five communities are described elsewhere [9]. The initial cohort in all the five communities was closed at 18 months. Approximately 3 months after all the volunteers had reached the end of the study, those from two of the five fishing communities (one each in Masaka and Wakiso) with the biggest population and relative easy accessibility were invited to return for an extended follow-up up to 30 months (two additional visits). At the same time the volunteers were requested to travel away from the fishing communities to attend their visits at the MRC/UVRI and UVRI/IAVI clinics located 10–40 km from the fishing communities. Similar data as collected in the initial follow up period was gathered. These clinics could be the nearest ideal trial sites for future efficacy trials rather than fishing communities which lack the infrastructure for such trials.

### Definitions

Fisher-folk are defined as not only fishermen but individuals living on the lakeshore who directly or indirectly derive their livelihoods from the fishing industry. These individuals include fish traders, fish processors, boat builders, families of fishermen, restaurant and bar workers, sex workers and others engaged in small scale businesses on the lakeshore. We defined study end point/completion as completing all the scheduled visits at 18 and 30 months or until an HIV infection occurred during follow-up. Study dropout was defined as missing any visit for any reason. Consistent condom use was considered to be use of condom at every sex act with every partner. Regular alcohol consumption was defined as consuming alcohol daily.

The number of sexual partners was dichotomized to none or one vs two or more. The length of time that the volunteer had spent at the fishing site was categorized as less than 1 year, 1–5 years and more than 5 years.

### Statistical analysis

Data were captured using MS Access and analyzed using Stata 11 [StataCorp, College station, Texas, United States of America (USA)]. The study fishing communities were denoted by the letters A, B, C, D and E in order to maintain confidentiality. We examined the association between volunteer dropout and the following independent variables; age (categorized as 13–24, 25–34 or 35–49), gender, fishing community, marital status, occupation, ethnicity, religion, highest educational level attained, how long the volunteer had lived at a given fishing community, whether or not the volunteer had been away from home for ≥2 nights in the month preceding the interview, number of sexual partners in the previous 3 months, having a new sexual partner (someone a volunteer had never previously engaged in sexual act with), having given or received gifts in exchange for sex, frequency of alcohol consumption, marijuana use, history of STIs and knowledge of having had sex with an HIV positive partner. Time to dropout was analyzed using discrete time survival models [13]. Appropriate variables were treated as time-varying. The association between cohort dropout and the potential explanatory factors was assessed by means of hazard ratios and 95 % confidence intervals. We defined a baseline hazard function as a piecewise linear function with one slope up to month 18 and a second slope from month 18 onwards. We further examined whether there was an interaction between each of the explanatory factors and time period (≤18 months or >18 months). Unadjusted (univariable) analyses were conducted and a likelihood ratio test (LRT) used to screen for variables to be included in the adjusted (multivariable) discrete time logit models. Factors that were significant at P < 0.05 in unadjusted analyses were selected for inclusion in the multivariable model. In the final multivariable model, variables that were not significant at P < 0.05 were removed from the model using a backward elimination approach. A separate (sensitivity) analysis was performed defining dropout as a volunteer enrolling in the cohort and not returning for any of the subsequent follow up visits as opposed to missing one visit for any reason after enrolment.

### Ethical considerations

The study was approved by the Uganda Virus Research Institute (UVRI) Science and Ethics Committee and Uganda National Council for Science and Technology. Written informed consent/assent was obtained from each volunteer. Study volunteers were provided with condoms, risk reduction counseling, outpatient medical care including diagnosis and treatment of STIs. Volunteers with incident HIV infections were referred for support and care, and offered enrolment in an ongoing Virology sub-study.

## Results

**Fig. 1:**
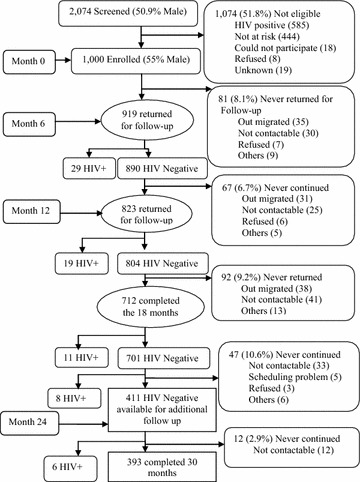
Study profile

A total of 2074 individuals (50.9 % men), median age of 28 years (Interquartile Range (IQR) = 23–34 years) were screened. Nearly half 1000 (48.2 %) fulfilled the eligibility criteria for joining the HIV incidence study cohort and were enrolled (Additional file 1: Table S1).Of those enrolled, 318 (31.8 %) were from site B and 259 (25.9 %) from C. The most frequent reasons for exclusion were HIV positive status 585 (54.4 %) or did not meet the criteria for risk 444 (41.3 %) (Fig. 1).

Enrolled volunteers were mostly men (55.0 %), aged below 34 years (78.2 %), of Christian faith (74.1 %), the majority had primary education (63.5 %) and were married (67.6 %). Most were engaged in fishing or fishing related occupations (36.7 %) or small scale businesses (29.6 %). They were mainly of non-Baganda ethnicity (53.3 %) and had lived in the fishing communities for more than 1 year (80.9 %). Compared to those that completed the study, dropouts were younger (median age of 27 vs. 29, P < 0.01), males (33.1 % vs. 26.0 %, p = 0.02), single (34.9 % vs. 27.5 %, p = 0.02), non-Baganda (33.5 % vs. 25.7 %, p = 0.01) and having lived in the fishing community for 0–1 year (29 % vs. 22.8 %, p < 0.01) but were otherwise similar in terms of other characteristics. In this cohort, HIV incidence rate of 4.9 (95 % CI 3.8–6.3) per 100 PYAR was recorded with 5.2/100 (3.7–7.3) and 4.5/100 (3.1–6.7) in men and women, respectively [9].

Figure 1 presents volunteer retention and reasons for dropping out of the cohort; 91.9, 85.2 and 76.0 % completed 6, 12 and 18 months of follow up respectively. Of the 577 volunteers enrolled from communities B and C, 466 (81 %) completed 18 months of follow up. These were contacted for additional follow up (24 and 30 months). Of the 466 contacted, 8 were found HIV+, 47 could not continue due to the following reasons (33-uncontactable 5-scheduling problems, 3-refused and 6-other). Therefore 411 HIV negative volunteers were available for months 24 follow up Fig. 1. Thus 456/577 (79 %) volunteers are regarded as having completed 24 months in the two communities including 45 that seroconverted before 24 months of follow up. Furthermore, a total of 393 in the two communities were seen at the month 30 visit including six that seroconverted between 24 and 30 months. Thus in total 444/577 (76.9 %) can be regarded as having completed the study at 30 months, including 51 (8.8 %) that seroconverted either before (37) or after (14) 18 months of follow up.

Overall, 299/1000 (29.9 %) volunteers missed at least one visit (dropped out of the study). The main reasons for volunteer dropout were migration and not being contactable Fig. 1. As shown in Additional file 1: Table S2, unadjusted factors associated with cohort dropout included gender (men; unadjusted hazard ratio (uHR) = 1.4, 95 % CI: 1.1–1.7), fishing community (with dropout highest in community E and lowest in community A), age group 13–24 years being more likely to dropout than those aged 25 or older, marital status [married; uHR = 0.7 (0.6–0.9)], ethnicity [non-Baganda; uHR = 1.4 (1.1–1.8)], time spent in the fishing community [0–1 year;uHR = 2.5 (1.8–3.5)], number of sexual partners [having two or more partners; uHR = 1.4 (1.1–1.8)], having a new sexual partner; uHR = 1.5 (1.2–2.0), being away from home for ≥2 nights; uHR = 1.4 (1.1–1.7), Marijuana use; uHR = 1.9 (1.4–2.8) and giving gifts in exchange for sex; uHR = 1.4 (1.1–1.8).

In the multivariable model, gender, age group, fishing community, ethnicity, time spent in the fishing community, having a new sexual partner, being away from home for ≥ 2 nights and marijuana use were independently associated with cohort dropout (Additional file 1: Table S3). Volunteers aged 13–24 years old [adjusted hazard ratio (aHR) = 1.3 (95 % CI: 1.0–1.9)] were more likely to dropout than those aged 25 or older. Volunteers who had stayed in the fishing communities for one year or less were 2.5 times more likely to dropout of the cohort [aHR = 2.5 (1.8–3.6)] compared to those that had stayed 5 or more years. Marijuana users were 1.7 times more likely to dropout; [aHR = 1.7 (1.2–2.5)]. Men were more likely to dropout [aOR = 1.4 (1.1–1.8)], non-Baganda were more likely to dropout than [aHR = 1.5 (1.2–1.9)], those having a new sexual partner; [aHR = 1.3 (1.0–1.7)] and those who were away from home for ≥2 nights [aHR = 1.4 (1.1–1.8)] and the risk of dropout also varied between the five communities. In the sensitivity analysis, similar predictors of dropout were identified using definition of dropout as missing subsequent follow up visits, however being away from home for ≥2 nights in the month preceding the interview and fishing community were no longer statistically significant.

## Discussion

We pre-selected a high risk group for this cohort study in which HIV incidence and other risky sexual behaviour were shown to be high [11]. Contrary to the perception that fisher-folk may be difficult to retain in longitudinal studies, we have demonstrated a modest study dropout in fishing communities comparable to that reported in neighbouring inland non-fishing communities [9, 10]. In addition high risk individuals have been associated with similar loss to follow-up in the neighbouring fishing 23.1 % [8] and non-fishing community 16.3 % [14].

The results suggest a number of factors that are associated with dropout from the fisher-folk cohort including young age, male gender, ethnicity, having a new sexual partner, time spent in the fishing communities, being away from home for ≥2 nights and marijuana use. Consistent with other studies in the neighbouring fishing [8] and non-fishing communities [9, 10], USA [15] and Brazil [16], study dropout was associated with age, with those in the youngest age group (13–24) being most likely to dropout. Since there were nearly equal proportions of men (49 %) and women (51 %) in this age group, this could be due to both looking for work and new relationships.

Dropping out was associated with the ethnicity of the volunteers, with non-Baganda being more likely to dropout. This has been indicated in the previous study further North of Lake Victoria [8]. The majority of non-Baganda volunteers come from further afield, including neighbouring countries (Kenya, Tanzania and Rwanda) and some are traders and transporters who might spend relatively long periods away from the fishing communities. These were more likely to be involved in fishing or related activities compared to the Baganda who were likely to be engaged in small scale businesses. Furthermore, number of years spent in the fishing site was independently associated with study dropout, with those who have spent more than 1 year in the fishing communities being less likely to dropout. This further demonstrates that individuals who have lived in a community for a long time are more likely to be available for recruitment in prevention studies. Additionally these could easily be retained in study follow up. The finding that dropout was associated with use of marijuana is consistent with findings from cohorts in the USA [17] and Japan [18], where similar dropout rates were observed among illicit drug users.

In this cohort we ascertained the frequency of reporting a new sexual partner for both men and women and this was independently associated with cohort dropout. This is consistent with the results from the inland non-fishing community in which the reported number of lifetime partners was higher in those who changed residence to other neighboring villages and there was also more risky sexual behavior reported among those who changed residence [14]. The fact that higher risk volunteers were more likely to drop out could lead to underestimation of HIV incidence in a prevention trial.

A major strength in terms of HIV prevention studies for these fishing communities compared to the general inland population is the high HIV incidence [11] and comparable dropout rates [9, 10]. Depending on the interventions being evaluated and other proven prevention strategies available, future efficacy trials in this population could require relatively shorter follow up period and a smaller sample size. A sensitivity analysis largely showed similar factors associated with dropout using a more liberal definition of dropout. It is encouraging to note that individuals from fishing communities can travel away from their work to attend their visits at clinic located further afield. In fact, our study showed minimal difference in dropout between clinic attendees at the fishing site and off site. This allays fears that their fairly good retention was only attained at study clinics located within the fishing communities with poor or non-existent infrastructure for conducting efficacy trials.

The limitations of this study include having obtained sexual risk behaviour data by self-report in a face to face interview which could have led to underreporting by women or over reporting by men depending on who is interviewing them [19]. The use of mobile phone short messaging service (SMS) has been shown to be more reliable in collecting self-reported sexual activity [20]. However, the mobile phone services are not well developed in some fishing communities with intermittent mobile network connections likely to disrupt the messaging service.

The most common reason for missing study visits was migration (Fig. 1). This has been associated with an increased risk of HIV infection probably due to higher risk sexual behaviour among those who move [14]. Therefore using recruitment strategies that avoids known predictors of volunteer missing visits would facilitate high retention. Alternatively strategies that aim at retaining volunteers who are likely to miss visits are needed to maximise retention.

## Conclusion

The dropout rates among the fishing communities along the shores of Lake Victoria are comparable to those of stable inland non-fishing communities which is contrary to the perception that fisher-folk are difficult to retain in follow-up studies because of their mobility. Volunteers from these communities demonstrated that they can still attend their scheduled follow up visits even when requested to attend a research clinic that is not located close to their fishing communities. In addition studies among fishing communities have demonstrated high HIV incidence. These suggest that HIV vaccine efficacy trials in this population may be feasible. However, targeted strategies aimed at recruiting and retaining volunteers from fishing communities are required. These could be in the form of regular counselling on attending study visits in combination with reminders by both telephone calls and home visits. Also it is important to note that future HIV vaccine trials would likely have more frequent study visits which could reduce the number of missed visits or dropouts. Therefore further research on study retention in a cohort with more frequent visits at a research clinic with the infrastructure suitable for HIV prevention trials are needed to reaffirm the suitability of fishing communities for intervention studies.

## Additional file

10.1186/s13104-015-1804-6 Screening, enrolment and follow up of fisher folks in rural and semi urban Uganda.

## Abbreviations

AIDS: acquired immunodeficiency syndrome
CI: confidence interval
EDCTP: European and developing countries clinical trials partnership
HIV: human immunodeficiency virus
HR: hazard ratio
IAVI: International AIDS vaccine initiative
LRT: likelihood ratio test
MRC: Medical research Council
SMS: short messaging service
STI: sexually transmitted infection
USA: United States of America
UVRI: Uganda virus Research Institute
VPS: vaccine preparatory studies
