# Supplementary material for: Factors associated with dropout in a long term observational cohort of fishing communities around lake Victoria, Uganda
Source: BMC Res Notes. 2015 Dec 24;8:815. doi: 10.1186/s13104-015-1804-6 (PMC4690385; doi:10.1186/s13104-015-1804-6)
Supplement: Supplementary file 1 — 10.1186/s13104-015-1804-6 Screening, enrolment and follow up of fisher folks in rural and semi urban Uganda. [file 13104_2015_1804_MOESM1_ESM.doc]

**Table 1: Background characteristics of** fisher folks in rural and semi urban Uganda

| Variable | Categories | Count | Percentage |
| --- | --- | --- | --- |
| Sex | Female | 450 | 45.0 |
|  | Male | 550 | 55.0 |
| Fishing community | A | 182 | 18.2 |
|  | B | 318 | 31.8 |
|  | C | 259 | 25.9 |
|  | D | 87 | 8.7 |
|  | E | 154 | 15.4 |
| Age group | 35-49 | 218 | 21.8 |
|  | 25-34 | 430 | 43.0 |
|  | 13-24 | 352 | 35.2 |
| Religion | Christian | 741 | 74.1 |
|  | Muslim | 228 | 22.8 |
|  | Other | 31 | 3.1 |
| Education | None | 104 | 10.4 |
|  | Primary | 635 | 63.5 |
|  | Post Primary | 261 | 26.1 |
| Marital status | Single | 324 | 32.4 |
|  | Married | 676 | 67.6 |
| Occupation | Fishing/fishing related | 367 | 36.7 |
|  | Farming | 103 | 10.3 |
|  | Bar | 154 | 15.4 |
|  | Small scale business | 296 | 29.6 |
|  | Unemployed | 59 | 5.9 |
|  | Other | 21 | 2.1 |
| Ethnicity | Baganda | 467 | 46.7 |
|  | Non Baganda | 533 | 53.3 |
| Time spent in the fishing site | >5 | 346 | 34.6 |
|  | >1 to 5 | 463 | 46.3 |
|  | 0-1 | 191 | 19.1 |

**Table 2: Unadjusted background** factors associated with dropout in a fisher folk cohort in rural and semi urban Uganda

| **Variable** | **Categories** | | **Dropout n (%)** | | **uHR(95%CI)** | | **LRT p-value** | |
| --- | --- | --- | --- | --- | --- | --- | --- | --- |
| Overall | - | | 299 (29.9) | | - | | - | |
| Sex | Female | | 117 (26.0) | | 1 | | 0.01 | |
|  | Male | | 182 (33.1) | | 1.4(1.1-1.7) | |  | |
|  |  | |  | |  | |  | |
| Fishing community | A | | 33 (18.1) | | 1 | | 0.02 | |
|  | B | | 102 (32.1) | | 1.3 (0.8-1.9) | |  | |
|  | C | | 92 (35.5) | | 1.4 (1.0-1.9) | |  | |
|  | D | | 20 (23.0) | | 1.2 (0.7-2.2) | |  | |
|  | E | | 52 (33.4) | | 2.1(1.3-3.3) | |  | |
|  |  | |  | |  | |  | |
| Age group | 35-49 | | 55 (25.2) | | 1 | | <0.01 | |
|  | 25-34 | | 120 (27.9) | | 1.1(0.8-1.6) | |  | |
|  | 13-24 | | 124 (35.2) | | 1.6(1.2-2.2) | |  | |
|  |  | |  | |  | |  | |
| Religion | Christian | | 222 (30.0) | | 1 | | 0.72 | |
|  | Muslim | | 65 (28.5) | | 1.0(0.8-1.3) | |  | |
|  | Other | | 12 (38.7) | | 1.3(0.7-2.4) | |  | |
|  |  | |  | |  | |  | |
| Education | None | | 32 (30.8) | | 1 | | 0.49 | |
|  | Primary | | 182 (28.7) | | 0.9(0.6-1.3) | |  | |
|  | Post Primary | | 85 (32.6) | | 1.0(0.7-1.5) | |  | |
|  | | |  | |  | |  | |
| Marital status | Single | | 113 (34.9) | | 1 | | 0.01 | |
|  | Married | | 186 (27.5) | | 0.7(0.6-0.9) | |  | |
| Occupation | Fishing/fishing related | | 124 (33.8) | | 1 | | 0.25 | |
|  | Farming | | 26 (25.2) | | 0.7(0.5-1.1) | |  | |
|  | Bar | | 48 (31.2) | | 0.9(0.7-1.3) | |  | |
|  | Small scale business | | 82 (27.7) | | 0.8 (0.6-1.1) | |  | |
|  | Unemployed | | 15 (25.4) | | 0.7 (0.4-1.2) | |  | |
|  | Other | | 4 (19.1) | | 0.5(0.2-1.3) | |  | |
|  |  | |  | |  | |  | |
| Ethnicity Baganda | | | 120 (25.7) | | 1 | | 0.01 | |
| Non Baganda | | | 179 (33.6) | | 1.4(1.1-1.8) | |  | |
|  | | |  | |  | |  | |
| Duration in the fishing site | >5 | 79 (22.8) | | 1 | | <0.01 | |  |
|  | >1 to 5 | 137 (29.6) | | 1.5(1.1-2.0) | |  | |  |
|  | 0-1 | 83 (43.5) | | 2.5(1.8-3.5) | |  | |  |

**Table 2 continued: Unadjusted time changing factors associated with dropout.**

| **Variable** | **Categories** | **uHR(95%CI)** | **LRT p-value** |
| --- | --- | --- | --- |
| Number of sexual partners | 0/1 | 1 | 0.01 |
|  | 2+ | 1.4 (1.1-1.8) |  |
|  |  |  |  |
| Having a new partner | No | 1 | <0.01 |
|  | Yes | 1.5 (1.2-2.0) |  |
|  |  |  |  |
| Frequency of condom use with new partner | Consistent | 1 | 0.13 |
|  | Inconsistent | 1.1 (0.7-1.9) |  |
|  | Never | 0.7 (0.4-1.3) |  |
|  |  |  |  |
| Being away from home for at least 2 nights | No | 1 | 0.01 |
|  | Yes | 1.4 (1.1-1.7) |  |
|  |  |  |  |
| History of drug use | No | 1 | <0.01 |
|  | Yes | 1.9 (1.4-2.8) |  |
|  |  |  |  |
| Receiving gift in exchange for sex | No | 1 | 0.70 |
|  | Yes | 1.1 (0.8-1.5) |  |
|  |  |  |  |
| Gift giving in exchange for sex | No | 1 | 0.03 |
|  | Yes | 1.4 (1.1-1.8) |  |
|  |  |  |  |
| Frequency of alcohol use | Never | 1 | 0.91 |
|  | Irregular | 1.0 (0.8-1.3) |  |
|  | Regular | 1.0 (0.7-1.6) |  |
|  |  |  |  |
| Genital discharge | No | 1 | 0.83 |
|  | Yes | 1.0 (0.7-1.3) |  |
|  |  |  |  |
| Genital sores | No | 1 | 0.43 |
|  | Yes | 0.9 (0.7-1.2) |  |
|  |  |  |  |
| Knowledge of HIV+ partner | No | 1 | 0.30 |
|  | Yes | 1.4 (0.8-2.5) |  |
|  |  |  |  |
| Previous history of STI | No | 1 | 0.40 |
|  | Yes | 0.9 (0.6-1.2) |  |

_u_HR- Unadjusted hazard ratio, LRT-likelihood ratio test, CI-confidence interval

**Table 3: Adjusted factors associated with dropout in a fisher folk cohort from Lake Victoria, Uganda**

| **Variable** | **Categories** | **uHR(95%CI)** | **LRT p-value** |  | **aHR(95%CI)** | **LRT p-value** |
| --- | --- | --- | --- | --- | --- | --- |
| Sex | Female | 1 | 0.01 |  | 1 | <0.01 |
|  | Male | 1.4(1.1-1.7) |  |  | 1.4(1.1-1.8) |  |
|  |  |  |  |  |  |  |
| Fishing community | A | 1 | 0.02 |  | 1 | <0.01 |
|  | B | 1.3 (0.8-1.9) |  |  | 0.7 (0.5-1.1) |  |
|  | C | 1.4 (1.0-1.9) |  |  | 0.8 (0.5-1.2) |  |
|  | D | 1.2 (0.7-2.2) |  |  | 1.2 (0.7-2.2) |  |
|  | E | 2.1(1.3-3.3) |  |  | 1.8 (1.1-2.8) |  |
|  |  |  |  |  |  |  |
| Age group | 35-49 | 1 | <0.01 |  | 1 | <0.01 |
|  | 25-34 | 1.1(0.8-1.6) |  |  | 1.0 (0.7-1.3) |  |
|  | 13-24 | 1.6(1.2-2.2) |  |  | 1.3 (1.0-1.9) |  |
|  |  |  |  |  |  |  |
| Ethnicity | Baganda | 1 | 0.01 |  | 1 | 0.01 |
|  | Non Baganda | 1.4(1.1-1.8) |  |  | 1.5 (1.2-1.9) |  |
|  |  |  |  |  |  |  |
| Time spent in the fishing site | >5 | 1 | <0.01 |  | 1 | <0.01 |
|  | >1 to 5 | 1.5(1.1-2.0) |  |  | 1.4 (1.1-1.9) |  |
|  | 0-1 | 2.5(1.8-3.5) |  |  | 2.5 (1.8-3.6) |  |
|  |  |  |  |  |  |  |
| Having a new partner | No | 1 | <0.01 |  | 1 | 0.04 |
|  | Yes | 1.5 (1.2-2.0) |  |  | 1.3 (1.0-1.7) |  |
|  |  |  |  |  |  |  |
| Being away from home for ≥2 nights | No | 1 | 0.01 |  | 1 | 0.02 |
|  | Yes | 1.4(1.1-1.7) |  |  | 1.4 (1.1-1.8) |  |
|  |  |  |  |  |  |  |
| Drug use | No | 1 | <0.01 |  | 1 | <0.01 |
|  | Yes | 1.9(1.4-2.8) |  |  | 1.7 (1.2-2.5) |  |

uHR, unadjusted hazard ratio; aHR, adjusted hazard ratio (All factors were adjusted gender, age, ethnicity, fishing community, marital status, occupation, number of sexual partners, having new partner, drug use, gift exchange, mobility and duration lived in the fishing site; CI, confidence interval; LRT, likelihood ratio test.
